# Food insecurity impacts neuroblastoma pathogenesis in murine xenograft tumor models

**DOI:** 10.1038/s42003-025-08678-5

**Published:** 2025-08-31

**Authors:** Keyonna Williams, Sahiti Chukkapalli, Biao Hu, Delawrence Sykes, Kimber Converso-Baran, Olivia Tussing, Anjalika Dandamudi, Payton Thayer, Kendal Donnelly, Samantha Kaminsky, Benjamin Curtis, Yatrik Shah, Kenneth Resnicow, Erika A. Newman, On behalf of the Project on Poverty in Neuroblastoma Evolution (PINE) Study Group, Keyonna Williams, Sahiti Chukkapalli, Erika A. Newman

**Affiliations:** 1 Department of Surgery, Section of Pediatric Surgery, The Rogel Cancer Center, The University of Michigan Medical School, Ann Arbor, MI USA; 2 Center for Health Inequities in Pediatric Surgery, The Section of Pediatric Surgery, The University of Michigan Medical School, Ann Arbor, MI USA; 3 Department of Biology, Berry College, Mount Berry, GA USA; 4 Physiology and Phenotype Center, Frankel Cardiovascular Center, The University of Michigan Medical School, Ann Arbor, MI USA; 5 Unit for Laboratory Animal Medicine, In-Vivo Animal Core, The University of Michigan Medical School, Ann Arbor, MI USA; 6 Department of Molecular and Integrative Physiology, The University of Michigan Medical School, Ann Arbor, MI USA; 7 Department of Pediatrics, The Rogel Cancer Center, The School of Public Health, The University of Michigan Medical School, Ann Arbor, MI USA

**Keywords:** Cancer models, Paediatric cancer

## Abstract

A recent landmark study by the National Cancer Institute’s Children’s Oncology Group identified an independent association between childhood poverty and increased mortality rates in children with neuroblastoma, beyond known biological, access, or treatment variables. Neuroblastoma, a prevalent extracranial nervous system tumor in children, is influenced by both biological and environmental factors. Recent studies suggest a direct link between poverty and poorer cancer survival outcomes, yet the biological mechanisms underlying this influence remain poorly understood. We hypothesize that socioeconomic stressors like food insecurity significantly impact neuroblastoma progression. To investigate this, we developed a murine model that simulates socioeconomic challenges by varying chow accessibility to mimic food insecurity. Our results demonstrate that food insecurity significantly accelerates neuroblastoma tumor growth and metastatic spread compared to controls. This exacerbation of tumor burden in food-insecure mice underscores the potential influence of socioeconomic stressors on cancer dynamics and challenges the traditional focus on genetic and biological factors alone. Our findings highlight the crucial role of socioeconomic factors in tumor biology and advocate for a more holistic approach that integrates socioeconomics and social determinants into therapeutic strategies.

## Abstract (web summary)

Mechanistic modeling of food insecurity reveals how chronic social stress accelerates neuroblastoma progression through tumor-intrinsic and microenvironmental changes in stress signaling.

## Introduction

Neuroblastoma, a cancer characterized by its origin within the sympathoadrenal lineage of neural crest cells, remains one of the deadliest childhood malignancies, accounting for a significant proportion of cancer deaths^1^. A recent landmark study by the National Cancer Institute’s Children’s Oncology Group (COG) identified an independent association between childhood poverty and increased rates of relapse and mortality in children with neuroblastoma, independent of known biological, access, or treatment variables^2^. This finding prompts an urgent need for new research methodologies to scientifically evaluate the consequences of poverty and socioeconomic deprivation alongside traditional translational oncology approaches.

Food insecurity, a common consequence of poverty, acts as a significant household-level economic and social stressor^3^. The resultant health consequences have been linked to altered immune defense and increased disease burden^3,4^. Specifically, food insecurity induces chronic stress responses, evidenced by persistent alterations in cortisol levels, a physiological correlate of exacerbated stress known to suppress immune function and potentially impact disease outcomes^5,6^. Chronic stress related to socioeconomic deprivation has been associated with neurological and endocrine dysregulation in cancer^7–9^. This relationship is further complicated by the effects of chronic stress on hormonal and metabolic pathways. Prolonged stress can dysregulate the hypothalamic-pituitary-adrenal (HPA) axis and elevate cortisol levels, fostering a systemic environment conducive to cancer progression through distinct regulatory pathways such as the PI3K/AKT/mTOR pathway^5,6,10–13^.

The mTOR pathway, in particular, plays a crucial role in regulating autophagy and cell proliferation by acting as a molecular sensor for nutrient deficiency, growth factor insufficiency, and hypoxia^14–17^.mTOR’s ability to balance cell survival and cell death highlights its importance in responding to both the nutritional state and the extracellular environment. Its involvement in various diseases, including cancers, makes it a focal point of interest for understanding the impacts of chronic nutritional stress on cancer development and progression^18,19^Although the mechanisms by which mTOR mediates these processes are complex and still under investigation, mTOR inhibitors are being explored as potential therapeutics to treat various cancers, further underscoring the need to elucidate the intricate interactions between chronic socioeconomic stress and cancer biology^19^.

Robust models that ethically replicate human socioeconomic stress and deprivation are essential for elucidating the molecular pathways influencing cancer outcomes. Researchers have demonstrated that certain aspects of social stress such as food scarcity, social defeat, and isolation can be effectively modeled in animal studies, showing comparable stress responses to those experienced by humans^20–23^. However, previous research has largely overlooked the potential biological consequences of these forms of social stress and their influence on tumor mechanics and metastasis. Our investigation aims to bridge this gap by focusing on the biological changes driven by food insecurity, a critical dimension of socioeconomic stress.

We have established a reliable murine model designed to mimic socioeconomic deprivation, offering a unique platform to study the biological impacts on cancer progression, using neuroblastoma as a disease model. By methodically imposing conditions of socioeconomic deprivation, such as food scarcity and uncertainty, we aim to uncover the mechanisms by which environmental deprivation influences tumor responses. Our central hypothesis is that socioeconomic deprivation including food insecurity plays a crucial role in the progression of neuroblastoma, impacting disease progression and survival outcomes.

Here, we present findings demonstrating that food insecurity significantly influences growth of neuroblastoma tumors. Our research aims to advance the understanding of cancer biology by incorporating a critical yet often overlooked element: the social determinants of health, using a robust murine model. By examining the interplay between traditional biological factors and socio- environmental influences, we aim to elucidate the specific mechanisms by which socioeconomic stressors impact cancer progression. This comprehensive approach will inform more effective interventions and policies, ultimately striving to achieve greater health for all.

## Results

### Intermittent chow scarcity alters feeding behaviors in neuroblastoma xenografted mice

In alignment with findings that link poverty and social factors like food insecurity to increased cancer mortality rates, our study investigated how a food scarcity model influences feeding behavior and tumor progression in vivo^24,25^. Utilizing an established mouse model with IMR-32 neuroblastoma cells, we replicated a validated murine food insecurity regimen to investigate its impact^25^. To examine the effects on feeding behavior, we imposed a controlled, variable feeding regimen on neuroblastoma xenografted mice. Preliminary observations confirmed that mice typically consumed 1.25 g of standard chow daily. Implementing our nutritional deprivation protocol, we varied food availability over four days, providing between 50% and 150% of standard intake followed by a three-day phase of replenishment with chow at 500% of baseline, mimicking episodic food availability characteristic of food insecurity (Table 1).

**Table 1:** Controlled feeding paradigm for modeling food insecurity in mice

Week of Protocol	Day 1	Day 2	Day 3	Day 4	Day 5	Day 6	Day 7
1	75%	150%	50%	125%	500%	500%	500%
2	125%	50%	150%	75%	500%	500%	500%
3	150%	75%	125%	50%	500%	500%	500%
4	50%	125%	75%	150%	500%	500%	500%

Outline of the weekly feeding schedule used to model intermittent food scarcity. Percentages represent the relative amount of standard chow provided each day based on the mouse’s baseline daily intake. Each week follows a distinct feeding pattern, alternating between restricted and surplus feeding days. Days 5–7 consistently provide 500% of baseline intake, mimicking compensatory overfeeding following periods of food restriction. Baseline food consumption was 1.25 g/mouse/day.

**Fig. 1:**
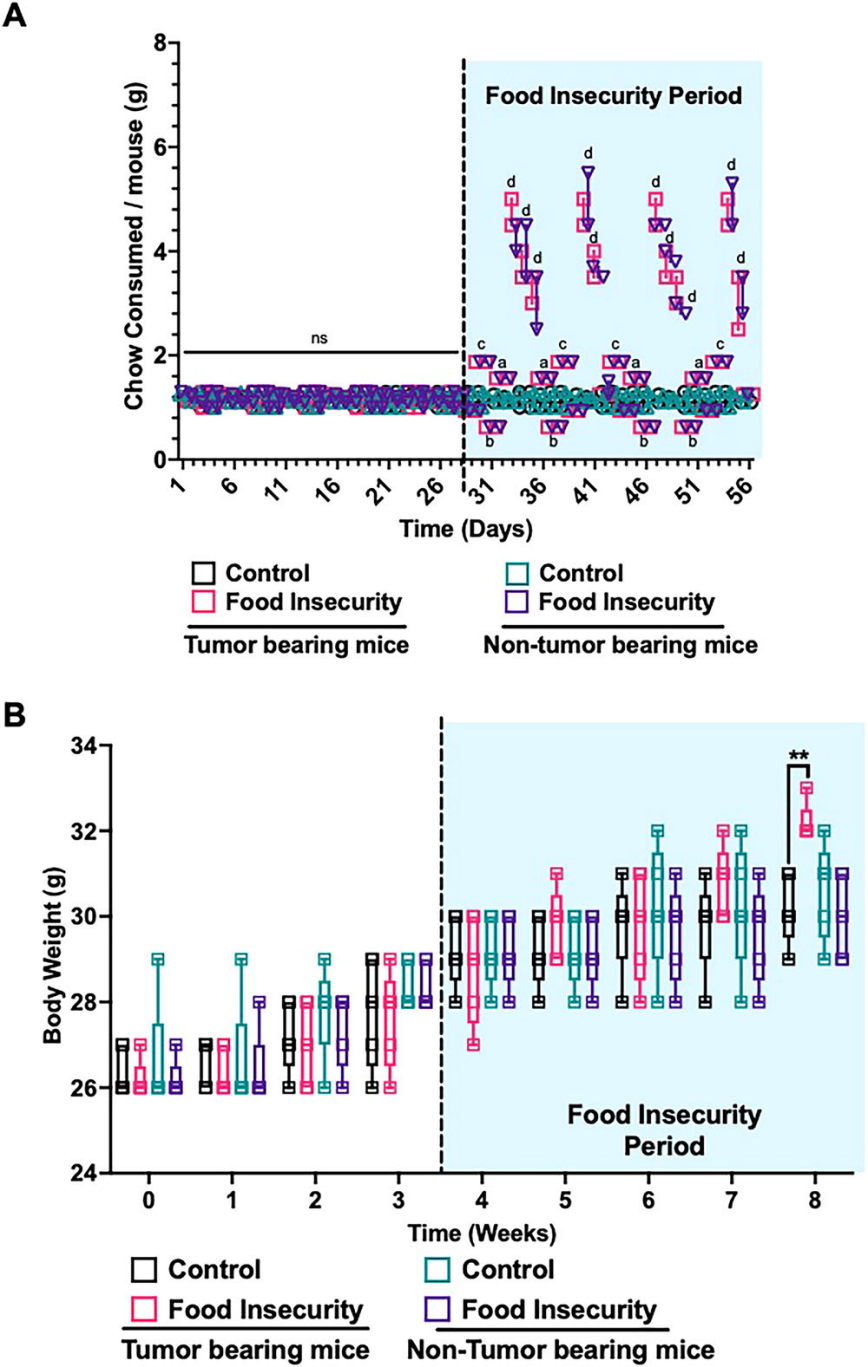
Intermittent chow scarcity induces altered feeding behaviors and hyperphagia in neuroblastoma xenografted mice. Chow consumption and body weight were monitored daily throughout the study to assess the physiological impact of intermittent food scarcity. **A** Mice subjected to intermittent chow availability exhibited binge eating behavior during refeeding periods, characterized by excessive chow consumption immediately following food restriction. This pattern was observed in both tumor-bearing and non-tumor-bearing groups, indicating a conserved response to dietary stress. **B** Body weight was tracked over time, with weekly measurements obtained alongside tumor volume assessments via high-resolution ultrasound. Food-insecure tumor-bearing mice demonstrated significant increase in body weight compared to controls, annotated a-d. (ns *p* > 0.05, a,*p < 0.05, b,**p < 0.01, c,***p < 0.001, d,****p < 0.0001).

During replenishment periods, mice in the food-insecure group exhibited significant hyperphagia, consuming up to three times more than their control counterparts. This hyperphagic response likely reflects a compensatory behavior driven by physiological and psychological adaptations to food scarcity, preparing for anticipated future deprivation. We did not observe differences in food consumption between non-tumor bearing and tumor bearing food insecure mice (Fig. 1A). By the experimental endpoint, tumor bearing food insecure mice showed an average weight gain of approximately 2 grams. This weight gain was primarily attributed to increased tumor mass rather than excess caloric intake, as the tumor weight in these mice increased more than tenfold, correlating with a significant rise in tumor burden compared to control mice. There were not any outliers of individual mice with higher weight gain. Simultaneously, no significant body weight changes were observed in control group food insecure mice that were not bearing xenografted tumors compared to mice that had *ad libitum* access to food (Fig. 1B).

### Intermittent chow scarcity modeling food insecurity accelerates tumor growth and enhances proliferation in neuroblastoma xenografts

**Fig. 2:**
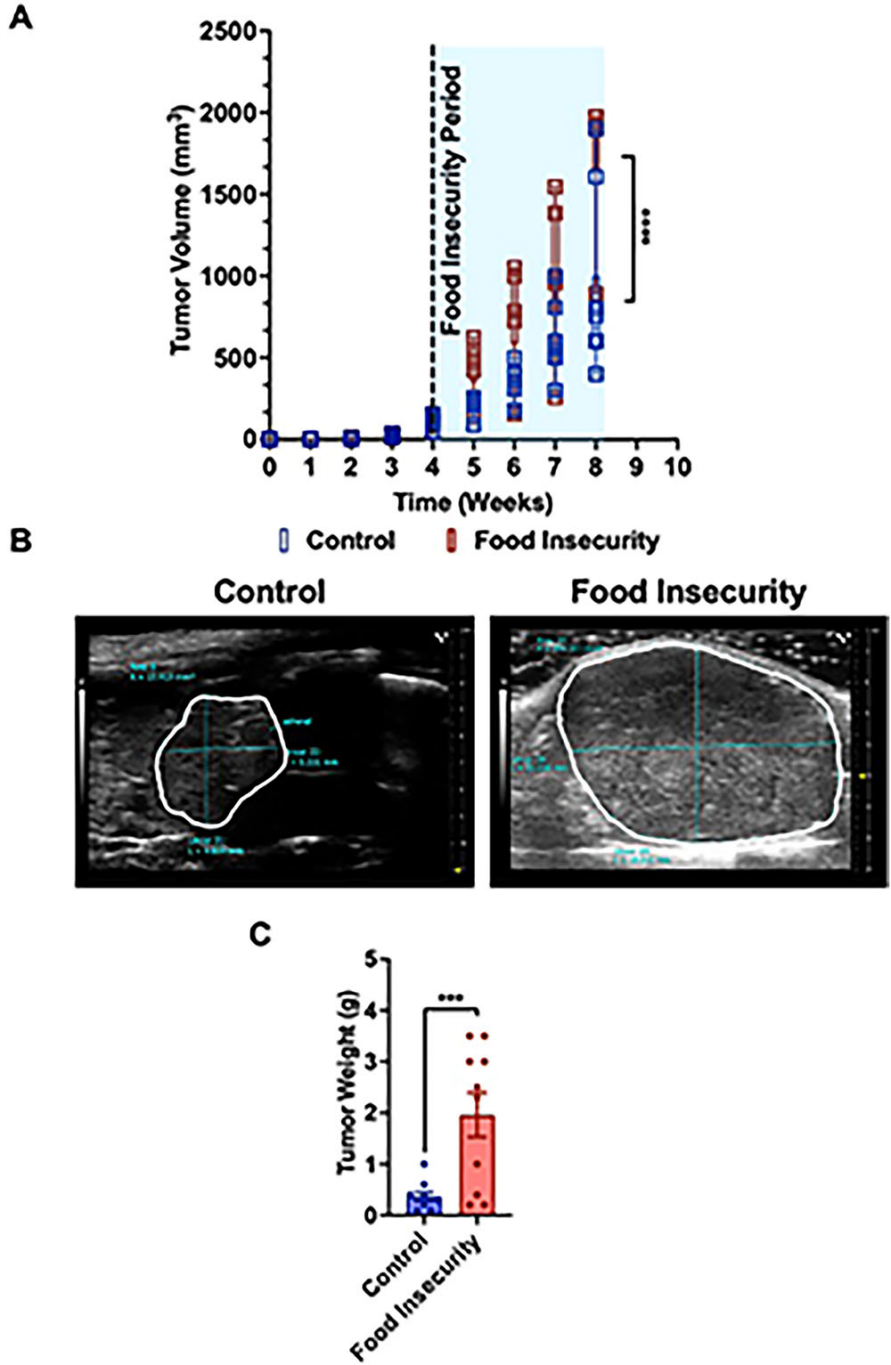
Intermittent chow scarcity, modeling food insecurity, accelerates tumor progression in neuroblastoma xenografted mice. **A** Tumor growth was measured longitudinally using ultrasound, revealing significantly larger tumors in the food insecurity group compared to controls. **B** Representative ultrasound images illustrate increased tumor size in food-insecure mice. **C** Harvested tumors from the food insecurity group weighed significantly more than those from the control group,further supporting the impact of food scarcity on tumor progression. (***p < 0.001, ****p < 0.0001).

Mice subjected to prolonged periods of limited food availability exhibited significant acceleration in tumor volume and size. Serial volume measurements with high resolution ultrasound imaging provided quantitative evidence of rapid tumor expansion (Fig. 2A, B). Ultrasound imaging confirmed significantly increased tumor burden within the food-insecure group, with pronounced visualization of larger volume tumors (Fig. 2B). Detailed necropsy analysis revealed that mice in the food-insecure group (*n* = 10) harbored tumors significantly larger in both volume and weight compared to xenografted tumors of their consistently nourished counterparts (*n* = 10) (Fig. 2C).

**Fig. 3:**
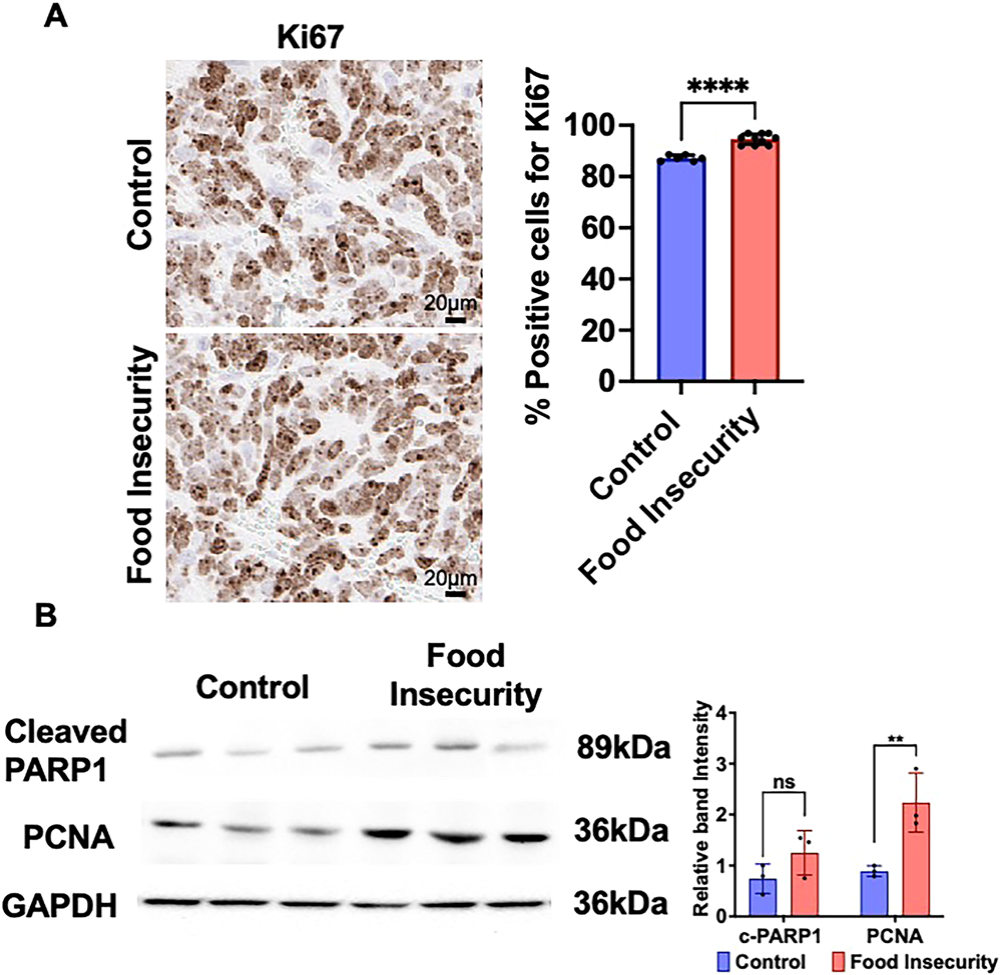
Intermittent food scarcity enhances tumor cell proliferation without affecting apoptotic markers. **A** Ki67 expression was assessed in tumor sections by immunohistochemistry. Tumors from the food-insecure group exhibited significantly higher Ki67 positivity compared to controls, indicating increased proliferative activity. **B** PCNA expression, another marker of cellular proliferation, was elevated in tumors from food-insecure mice compared to controls, further supporting enhanced tumor growth under conditions of dietary stress. In contrast, cleaved PARP1 expression, a key apoptotic marker, remained unchanged between groups. (ns, *p* > 0.05, *p < 0.05, **p < 0.01, ***p < 0.001, ****p < 0.0001).

Next, we assessed tumor cell proliferation by analyzing Ki67 expression, a marker of cell proliferation, using immunohistochemical staining and digital slide scanning. Tumors from the food-insecure group exhibited an average of 95% Ki67-positive cells, significantly higher than the 87% observed in control group tumors (Fig. 3A). To further evaluate proliferation, we examined PCNA (proliferating cell nuclear antigen) expression by immunoblotting, which revealed increased PCNA levels in tumors from food-insecure mice compared to controls (Fig. 3B). The impact of food insecurity on tumor cell death was investigated by analyzing apoptotic markers via immunoblotting and RT-PCR. No significant differences were observed in the expression of cleaved PARP1 between groups (Fig. 3B). These findings demonstrate that intermittent food scarcity, modeling food insecurity, significantly accelerates neuroblastoma tumor progression by enhancing tumor proliferation without increasing apoptotic cell death. Increased expression of Ki67 and PCNA in tumors from food-insecure mice further supports a proliferation-driven mechanism underlying tumor growth in this model.

Collectively, these results demonstrate a strong link between experimental food insecurity, disrupted feeding behaviors, and accelerated neuroblastoma tumor growth in vivo. This study underscores the profound tumor response to food scarcity and highlights the urgent need for further exploration into the biological mechanisms by which socioeconomic factors like food insecurity may drive tumor growth.

### Intermittent chow scarcity modeling food insecurity augments metastatic potential

**Fig. 4:**
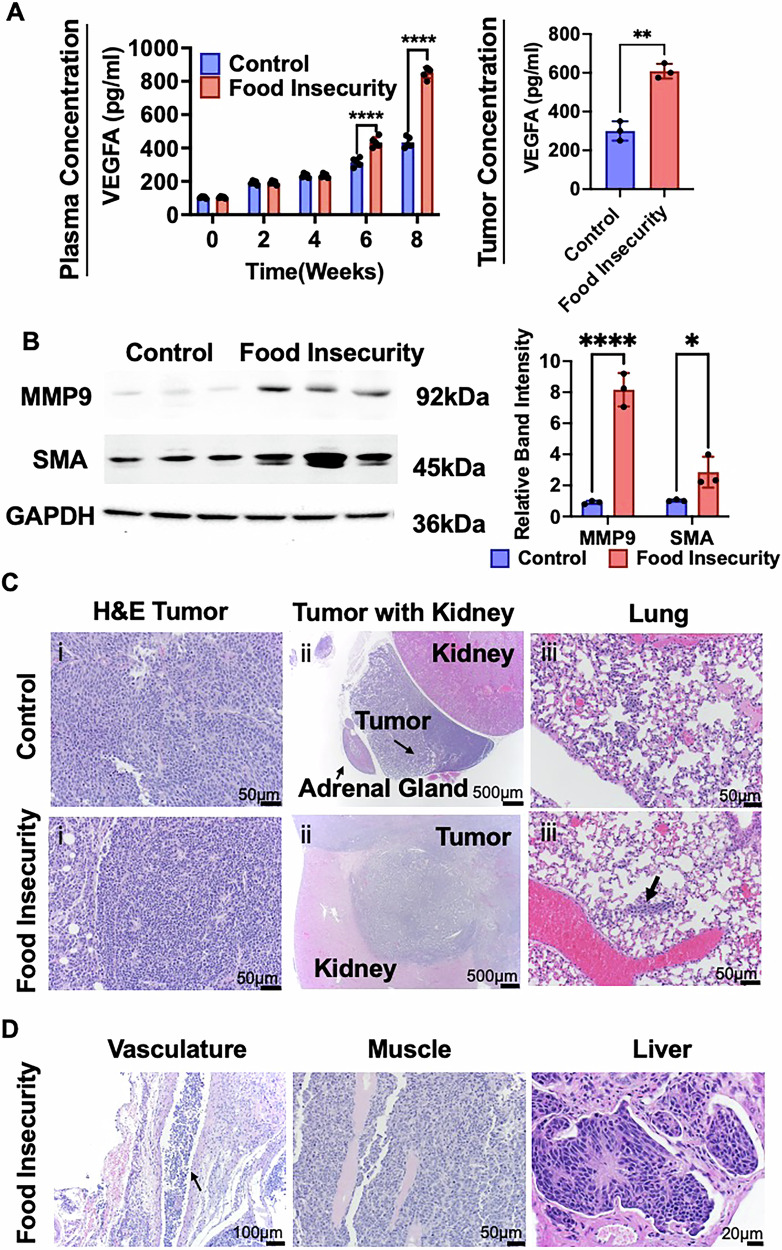
Intermittent chow scarcity augments metastatic potential. **A** VEGFA levels assessed from peripheral blood samples and harvested tumors of experimental mice. VEGFA levels were higher in the chow scarcity food insecurity modeling group compared to control groups at 6 and 8 weeks. **B** Western blots showing elevated levels of MMP9 and SMA in food insecurity group tumors, **C** (i)Primary tumor microscopy demonstrating characteristic small round blue neoplastic cells arranged in islands and rosettes with multiple mitotic figures (200x). (ii) Primary tumor (neoplastic small round blue cells) invading into adjacent kidney parenchyma in food insecure group (20x). (iii) Micrometastasis detected in lung tissue histologic analysis from food insecure group (×400). **D** Invasive tumor cells with local invasion into smooth muscle (×200), adjacent vasculature (×100) and liver (×200) from the food insecure group. (ns, *p* > 0.05, *p < 0.05, **p < 0.01, **p < 0.001, ****p < 0.0001).

To investigate the role of food insecurity in tumor vascularization, we first assessed VEGF expression as a key regulator of angiogenesis. Mice subjected to intermittent chow scarcity exhibited significantly elevated plasma VEGF levels compared to controls (Fig. 4A). To determine whether this systemic increase corresponded with local tumor VEGF expression, we further analyzed VEGF levels within tumor tissue. Consistently, VEGF expression was markedly higher in tumors from food-insecure mice (Fig. 4B), suggesting a tumor-intrinsic response to chow deprivation.

We further investigated the expression of key metastatic and invasive markers, matrix metalloproteinase 9 (MMP9) and alpha-smooth muscle actin (SMA) in harvested tumors to assess the impact of food insecurity on tumor aggressiveness. The expression of MMP9, a critical enzyme involved in extracellular matrix degradation and metastatic progression, was increased in tumors from the food-insecure group compared to controls, suggesting enhanced metastatic potential (Fig. 4B). Similarly, SMA expression was significantly elevated in tumors from food-insecure mice, indicating a more invasive tumor phenotype (Fig. 4B). These findings suggest that intermittent food scarcity promotes a pro-metastatic and invasive tumor microenvironment, potentially facilitating tumor dissemination and progression.

Comprehensive necropsy and histopathological analyses revealed a striking increase in tumor invasiveness and metastatic dissemination in food-insecure mice compared to controls. A substantial proportion of mice subjected to intermittent food scarcity (9 out of 10) exhibited direct tumor infiltration into the vasculature, a hallmark of aggressive disease progression. Additionally, local tumor spread into the kidney and adjacent tissues was observed in 6 and 9 out of 10 food-insecure mice respectively, a significantly higher incidence than in control animals, where such invasion was not detected (Fig. 4C, D). Beyond local invasion, intermittent food scarcity also drove increased systemic dissemination of neuroblastoma cells. Lung micrometastases were present in 5 out of 10 mice in the food-insecure group, a fivefold increase compared to control xenografted mice, where lung metastases were observed in only 1 out of 10 animals. Liver metastases were detected exclusively in food-insecure mice (2 out of 10), further supporting the association between food scarcity and enhanced metastatic potential (Fig. 4C, D). A comprehensive summary of metastatic incidence across experimental groups is provided in Table 2.

**Table 2:** Local invasion and distant metastatic characteristics

Characteristic	Control group	Food-insecure group
**Distant metastasis**		
Lung Metastases	Present (*n* = 1)	Present (*n* = 5)
Lung: Frequency of Metastatic Foci	Rare	Common
Lung: Size of Metastatic Foci	Small (*n* = 1)	Medium (*n* = 2), Large (*n* = 3)
Lymph Node Metastases	Not present	Present (*n* = 1)
Liver Metastases	Not present	Present (*n* = 2)
**Local tumor invasion**		
Kidney Infiltration	Not present	Present (*n* = 6)
Musculature Invasion	Not present	Present (*n* = 9)
Vascular Invasion	Not present	Present (*n* = 9)

Histopathological analysis of xenografted neuroblastoma tumors was conducted. This analysis revealed significant differences between the control and food-insecure groups. Lung metastases were more frequent and exhibited larger foci in the food-insecure group compared to controls. Additionally, lymph node and liver metastases were detected exclusively in the food-insecure group. Local tumor invasion was markedly increased in food-insecure mice, with significant infiltration into the kidney, musculature, and vasculature, whereas no such invasion was observed in control animals. These findings suggest that intermittent food scarcity promotes a more aggressive tumor phenotype with enhanced metastatic potential and local invasiveness.

These findings provide compelling evidence that intermittent food scarcity profoundly exacerbates neuroblastoma tumor aggression by promoting local invasion and increasing the likelihood of distant metastasis. This study underscores the potential impact of variable food availability as a tumor-promoting stressor, with important implications for understanding the biological mechanisms linking social adversity to cancer progression. Further investigations into the molecular pathways underlying these effects are warranted, with the ultimate goal of identifying therapeutic strategies to mitigate the influence of environmental stressors on tumor behavior.

### Food insecurity-induced neuroendocrine stress enhances neuroblastoma growth

**Fig. 5:**
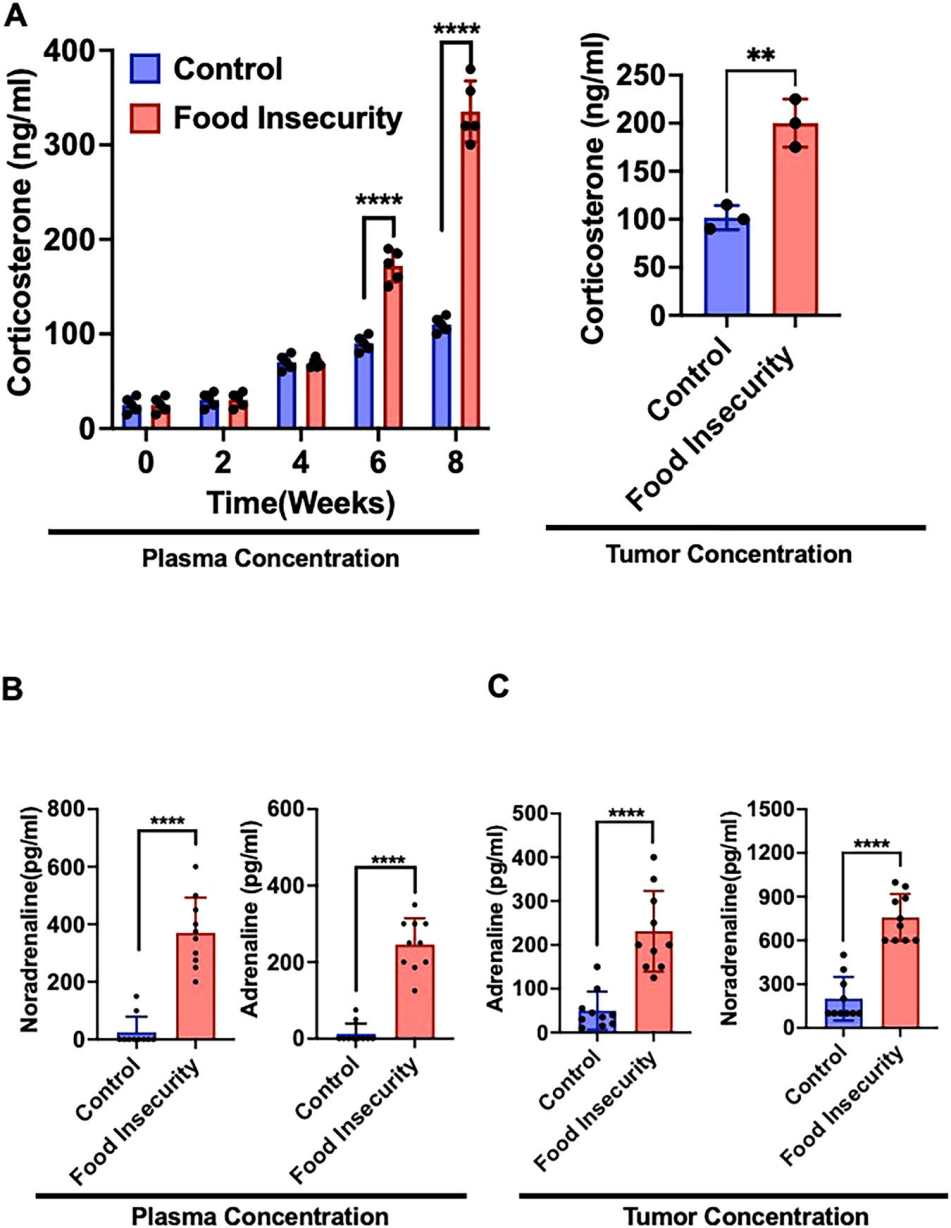
Food insecurity elevates systemic and intratumoral stress hormone levels. Corticosterone (HPA axis activation marker) and catecholamines (sympathetic neurotransmitters) were measured pre- and post-food insecurity. **A** Plasma and tumor corticosterone levels were significantly elevated in the food-insecure group compared to controls, indicating a heightened systemic stress response. **B**, **C** Catecholamine levels were assessed in both plasma (systemic) and tumor tissue (intratumoral). Food insecurity resulted in a significant increase in both systemic and intratumoral catecholamine levels, suggesting enhanced sympathetic activation within the tumor microenvironment. (**p < 0.001, ***p < 0.001, ****p < 0.0001).

Our study assessed the neuroendocrine effects of food insecurity on neuroblastoma by monitoring critical stress hormones in xenografted mice subjected to varying food availability. Corticosterone, the primary stress hormone in rodents, was evaluated as a stress marker. Biweekly plasma evaluations showed a substantial increase in corticosterone levels in food-deprived mice, significantly exceeding the elevated levels observed in the control group (Fig. 5A). Analysis of the xenografted tumors also revealed elevated corticosterone (Fig. 5A) and catecholamine levels, specifically adrenaline and noradrenaline in food-insecure mice, indicating heightened neuroendocrine stress (Fig. 5B, C). This hormonal dysregulation suggests a systemic stress response induced by food insecurity, potentially contributing to an environment conducive to tumor growth. These findings highlight that socioeconomically driven stressors like food insecurity may lead to intrinsic biological changes that may influence disease progression in cancer.

### Modeling food insecurity activates tumor survival pathways

Given the well-documented effects of dietary stress on behavioral and hormonal regulation, we investigated genes associated with the hypothalamic-pituitary-adrenal (HPA) axis and neurotransmitter signaling in response to food insecurity. A neurotransmitter PCR array revealed significant downregulation of key GABAergic pathway genes (*GABRG2, GABRA5, GABBR1, GABR4*, and *GABRB1*) in tumors from food-insecure mice. While GABAergic signaling is traditionally associated with the central nervous system, it also plays a functional role in peripheral tumor biology. In non–small cell lung cancer models, stress-induced suppression of intratumoral GABA enhances β-adrenergic signaling and tumor growth effects that are reversed by GABA administration, independent of central nervous system activity^12^. Consistent with this, we observed downregulation of GABA pathway genes within neuroblastoma xenografts under food insecurity, supporting a tumor-intrinsic, peripheral role for GABA in modulating stress-driven growth. GABAergic signaling has a crucial role in reducing anxiety and stress, its suppression suggests that modeling food insecurity induces a stress-associated neurochemical shift.

**Fig. 6:**
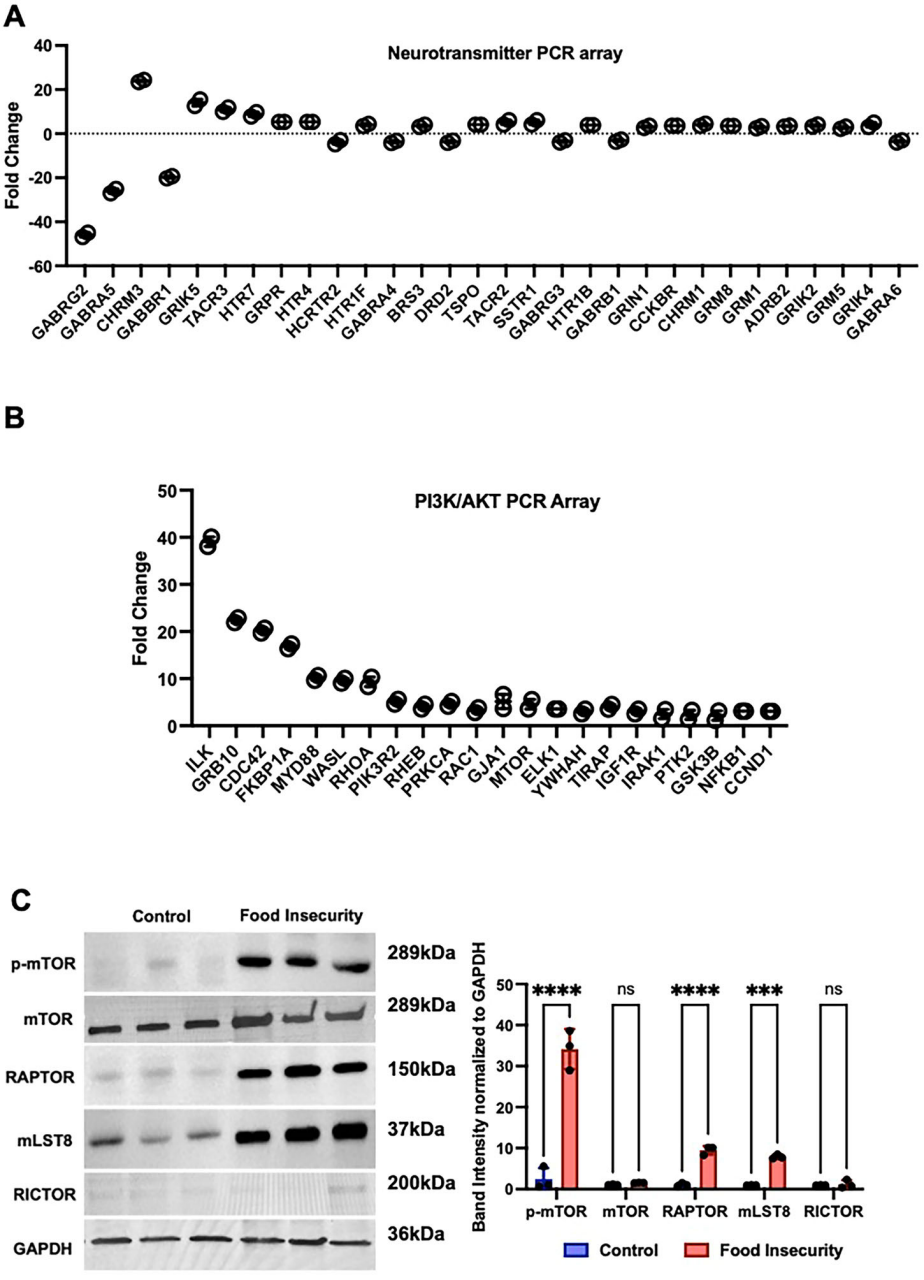
Food insecurity induces stress-responsive neurotransmitter shifts and activates PI3K/AKT/mTORC1 signaling in neuroblastoma. **A** Neurotransmitter signaling genes exhibiting ≥3-fold differential expression in tumors from food-insecure mice compared to controls are shown. Downregulation of GABAergic components (*GABRG2, GABRA5, GABBR1*) and upregulation of stress-associated receptors (*CHRM1, CHRM3, ADRB2*) reflect a stress-adaptive transcriptional shift. **B** Genes within the PI3K/AKT pathway showing ≥3-fold upregulation in tumors from food-insecure mice include key regulators of tumor survival and metabolic adaptation (*ILK, PIK3R2, AKT3, MTOR, IGF1R*), indicating coordinated activation of pro-survival signaling. **C** mTORC1 pathway activation was confirmed at the protein level by immunoblot analysis. Representative blots and quantification demonstrate increased expression and phosphorylation of mTORC1 complex components (mTOR, RAPTOR, GβL), consistent with metabolic reprogramming in response to dietary stress. Only statistically significant changes (*p* < 0.05) are displayed in panels (**A**, **B**). (ns *p* > 0.05, ***p < 0.001, ****p < 0.0001).

Beyond the GABAergic system, additional neurotransmitter-related genes involved in memory, synaptic plasticity, and stress response (*GRIK4, GRIK2, GRM6, GRM3, HTR7, HTR1D*, and *HTR2A*) were deregulated in tumors from food-insecure mice. Muscarinic acetylcholine receptors (*CHRM3* and *CHRM1*), along with β-adrenergic receptor ADRB2, were upregulated in response to food insecurity. Notably, these receptors are known to activate the PI3K/AKT signaling cascade through Gq-mediated pathways, which play a central role in tumor progression, angiogenesis, invasion, metastasis, and immune modulation (Fig. 6A).

To further investigate the molecular adaptations induced by food insecurity, we examined key regulators of the PI3K/AKT pathway, a major signaling axis governing cell proliferation, survival, and migration. Multiple genes associated with tumorigenic signaling, including *ILK, GRB10, CDC42, FKBP1A, MYD88, WASL, RHOA, PIK3R2, MTOR, IGF1R*, and *AKT3*, were significantly upregulated in tumors from food-insecure mice (Fig. 6B). These findings suggest that food insecurity activates a pro-survival and pro-metastatic transcriptional program that enhances tumor adaptability in response to environmental stress.

At the protein level, Western blot analyses revealed a pronounced increase in RAPTOR expression in tumors from food-insecure mice, suggesting preferential activation of the mTORC1 pathway, a key regulator of cellular metabolism and growth. Notably, genes specific to the mTORC1 signaling pathway were significantly upregulated in tumors from food-insecure mice. Among them, integrin-linked kinase (ILK) exhibited the largest fold change. ILK plays a critical role in neuroblastoma proliferation and survival by regulating PTEN, a key tumor suppressor involved in PI3K/AKT signaling^26^. Given the pronounced upregulation of ILK and other mTORC1-related genes in the food-insecure group, we conducted a more in-depth investigation into the mTORC1 pathway, aiming to elucidate its role in tumor adaptation to environmental stress. This upregulation was accompanied by increased phosphorylation of mTOR kinase and its essential cofactor GβL/mLST8 (mammalian lethal with SEC13 protein 8), confirming hyperactivation of mTORC1 signaling (Fig. 6C). Notably, RICTOR expression, a defining component of the alternative mTORC2 complex, remained unchanged, highlighting a selective activation of mTORC1 rather than mTORC2 in response to food scarcity-induced tumor adaptation. These findings suggest that food insecurity drives a tumor-specific metabolic reprogramming that enhances survival under stress conditions. mTORC1 expression and activity are known to fluctuate in response to both nutrient availability and circadian rhythms. To control for these variables, all samples were collected at a consistent time point (08:00 am) following a standardized three-day ad libitum feeding period.

Collectively, these results provide strong evidence that a socioeconomic stressor, food insecurity, activates oncogenic survival pathways in neuroblastoma through a coordinated upregulation of stress-responsive neurotransmitter signaling and PI3K/AKT/mTORC1 activation. The convergence of elevated stress hormone signaling with selective mTORC1 activation mirrors established literature linking chronic stress to oncogenic pathway modulation^27^. While this study was designed to model the physiological effects of food scarcity rather than directly manipulate mTORC1, our findings suggest that stress-induced metabolic shifts contribute to its activation. Future investigations will explore whether this activation is primarily mediated by elevated stress hormones or a direct response to nutrient deprivation. These findings underscore the potential for further investigation into mTORC1-targeted therapeutic strategies to mitigate the adverse impact of socioeconomic stress on neuroblastoma tumor progression.

## Discussion

This study provides compelling evidence that chow scarcity, a model of socioeconomic deprivation, significantly impacts the growth and metastatic progression of neuroblastoma in vivo. By integrating behavioral, hormonal, and molecular analyses, our findings shed light on the complex interplay between external socioeconomic factors and intrinsic biological mechanisms driving tumor growth, survival, and metastasis. Our findings reveal new perspectives on the established links between stress, endocrine responses, and cancer biology, suggesting avenues for therapeutic intervention and public health strategies.

The observed hyperphagic behavior in food-insecure mice suggests an adaptation to intermittent food availability, reflecting a biological strategy to mitigate the impact of food scarcity. This compensatory overconsumption could potentially alter metabolic pathways that may support tumor growth. Our data underscores the link between dietary stress and tumor burden, highlighting the need for nutritional considerations in cancer management. It also suggests that periods of nutritional stress followed by refeeding could create metabolic conditions that favor tumor growth, an area that warrants further exploration.

The significant elevations in corticosterone, adrenaline, and noradrenaline levels in food-insecure mice establish a clear link between socioeconomic stress and neuroendocrine disruption. This response was seen as early as week six. These hormones are known to influence a variety of pathways associated with cell proliferation and survival, suggesting that chronic stress creates a pro-tumorigenic environment^7,28^. The systemic stress responses observed in this study underscore the importance of addressing chronic stress in cancer patients, as unmanaged stress could potentially worsen their prognosis by creating conditions that favor tumor progression and metastasis.

Genetic heterogeneity of neuroblastoma remains an area of active investigation, with transcriptomic analyses identifying over 100 genes associated with aggressive neuroblastoma subtypes through clustering analyses of high-risk tumor samples^29^. PCR analysis of tumors from both our experimental control and food-insecure groups identified a gene expression profile consistent with molecular signatures associated with high-risk neuroblastoma. However, tumors from the food-insecure group exhibited a distinct transcriptional shift characterized by the upregulation of genes involved in oncogenic signaling, metabolic adaptation, and survival pathways. Notably, genes specific to the mTORC1 signaling cascade were significantly overexpressed, suggesting that food insecurity may drive tumor progression through metabolic and stress-adaptive mechanisms.

The hyperactivation of the mTORC1 signaling pathway in response to food insecurity provides insights into the molecular adaptations that facilitate tumor survival and growth under stress. mTORC1 is a crucial regulator of cellular metabolism, growth, and survival, and its specific activation under conditions of food scarcity highlights its role as a key mediator in stress-induced tumor biology^17,18^. This pathway’s sensitivity to nutrient availability positions it as a potential target for therapeutic intervention, particularly in patients experiencing significant socioeconomic stressors^19^. Our findings support the hypothesis that targeting mTORC1 and its associated signaling cascades may mitigate the adverse effects of socioeconomic stress on tumor progression.

Children from low-income households with high-risk neuroblastoma treated with targeted immunotherapy experience significantly worse event-free survival (EFS) and overall survival (OS), as highlighted by Bona et al. While treatment protocols remain standardized across socioeconomic groups, disparities in outcomes persist, suggesting that non-biological stressors such as poverty, food insecurity, and chronic psychosocial adversity may have direct biological consequences on tumor progression and therapy resistance. One potential mechanistic link between socioeconomic disadvantage and worse neuroblastoma outcomes is mTORC1 hyperactivation, a pathway known to integrate nutritional, hormonal, and metabolic stress signals into pro-survival and pro-tumorigenic responses.

Thus, integrating mTORC1 inhibition as a therapeutic strategy in socially disadvantaged patients may hold promise for mitigating the biologically exacerbated disparities observed in neuroblastoma and potentially other cancers with disparate outcomes. Given the established role of mTORC1 inhibitors in sensitizing tumors to immunotherapy and chemotherapy, future investigations should explore whether targeting mTORC1-driven metabolic and immune dysregulation can help reduce the survival gap associated with poverty in pediatric oncology.

From a public health perspective, these results emphasize the critical role of socioeconomic factors in cancer biology. Policies that address food insecurity and socioeconomic disparities are paramount, as they not only improve general health but could also directly impact cancer outcomes. These insights highlight the need for holistic cancer care approaches that incorporate both medical treatments and social support systems.

The mouse model used in this study provides a controllable and ethical means to simulate socioeconomic stress, an approach that is not feasible in human studies due to ethical constraints. This model allows for precise manipulation of food availability and systematic analysis of biological responses, offering valuable insights that are difficult to obtain through observational studies in humans. Despite the robust findings, several limitations warrant consideration. The use of a murine model, while informative, may not fully capture the complexity of human socioeconomic stress and its implications. Human socioeconomic factors cannot be fully replicated in animal models, necessitating cautious extrapolation to clinical settings. Additionally, the study focuses solely on neuroblastoma, and the effects observed may vary across different cancer types. Future research should include clinical studies to validate these preclinical results in human populations and extend investigations to other cancers. It is important to acknowledge that mTOR pathway activity and systemic stress hormone levels are subject to circadian variation, which may influence experimental readouts. mTORC1 signaling is dynamically regulated by nutrient availability and circadian cues, and stress hormones such as corticosterone and catecholamines fluctuate across the diurnal cycle. To mitigate this variability, all samples in our study were collected at a standardized time point (8:00 am) following a three-day ad libitum feeding phase. While this approach minimized temporal confounders, we recognize that future studies incorporating longitudinal sampling and circadian-controlled conditions will be necessary to fully resolve the temporal dynamics of stress-responsive tumor signaling. Further, exploring the interplay between different socioeconomic stressors and biological responses could provide a deeper understanding of how various factors combined impact cancer progression. Investigating the potential for interventions that target stress pathways, such as mTORC1 inhibitors, in clinical settings remains a critical next step.

Given these results, several immediate steps can be taken to positively impact cancer patients facing socioeconomic stress and food insecurity:

1. Nutrition and Stress Management Programs: Healthcare providers should integrate nutrition and stress management programs into cancer care. Offering counseling on dietary practices, providing access to nutritious food, and incorporating stress-reduction techniques could mitigate the physiological impact of food insecurity and chronic stress.
2. Screening for Socioeconomic Stressors: Routine screening for socioeconomic stressors, including food insecurity, should be implemented during patient assessments. Identifying patients at risk can help tailor interventions and provide necessary support early in the treatment process.
3. Holistic Treatment Plans: Developing holistic treatment plans that address both medical and socioeconomic needs is crucial. Multidisciplinary teams, including social workers, nutritionists, and mental health professionals, should collaborate to provide comprehensive care that tackles the multifaceted challenges faced by socioeconomically disadvantaged patients.
4. Policy Advocacy: On a broader scale, advocating for policies that reduce food insecurity and socioeconomic disparities is essential. Supporting initiatives that provide food assistance programs, healthcare access, and financial support for low-income families can contribute to better health outcomes and potentially reduce cancer progression rates associated with socioeconomic stress.

Our study underscores a significant relationship between socioeconomic stress, modeled through food insecurity, and neuroblastoma progression. By highlighting the complex interplay between social determinants and cancer biology, we stress the urgent need for a more holistic approach to cancer care. Addressing both the biological and socioeconomic dimensions of health can lead to more effective treatment strategies, paving the way toward health equity for all.

## Materials and methods

### Cell lines

IMR-32 cells were obtained from ATCC in March 2023. Cells were maintained in minimal essential medium, supplemented with 10% FBS, 2 mmol/L glutamine, 100 U/ml penicillin, and 100 µg/ml streptomycin, 1 mmol/L pyruvate and 0.075% NaHCO3 at 5% CO2 incubator at 37 °C. Cells were maintained in culture for up to 20 passages and tested for mycoplasma at regular intervals.

### Mice protocol

Male immunocompromised NOD-SCID gamma (NOD.Cg-PrkdcSCIDII2rgtm1Wjl/SzJ) mice were purchased from The Jackson Laboratory (Wilmington, MA) between 4-6 weeks of age, and were housed five per cage with food and water ad libitum on a 12:12 light/dark cycle. Mice were given enviropak for enrichment. Mice were allowed to adjust to the new environment for two weeks before experiments were initiated. Experimental protocols were approved by the institutional animal care and use committee (IACUC) at University of Michigan, Ann Arbor, Michigan under protocol number PRO00011833. The rationale for first studying male mice was based on prior research suggesting that male mice exhibit heightened sensitivity to stress responses, particularly in models of depression and anxiety-related behaviors^30^. Given that our study aims to investigate the impact of food insecurity as a chronic stressor and its effects on tumor progression, we selected male mice to minimize potential variability introduced by sex differences in stress resilience and hormonal fluctuations. We fully acknowledge that sex is a critical biological variable, and future studies will incorporate both male and female cohorts to examine potential sex-specific differences in neuroblastoma adaptation to socioeconomic stressors. We have complied with all relevant ethical regulations for animal use.

### Tumor inoculation and monitoring

IMR-32 cells in the exponential growth phase were prepared for injection. 1 × 10^5^ IMR-32 cells suspended in 1:1 dilution of RPMI (Corning) and Matrigel (Corning) were percutaneously injected into the left adrenal gland of NSG mice using ultrasound guidance as previously described^24^. Tumors were monitored weekly for engraftment and growth using ultrasound. Tumor volumes were graphed using ½(ab2) formula. The maximum tumor size allowed by the IACUC is 2 cm in any one direction or 2000 mm³ in volume, and in none of the experiments were these limits exceeded.

### Chow scarcity food insecurity modeling protocol

The following in vivo food insecurity protocol was adapted from Estacio et al. for neuroblastoma xenografted mice^25^. Following tumor engraftment verification through serial ultrasound and volume measurements, xenografted mice were randomly assigned to either the food insecurity or control groups. The food insecurity group consisted of two cages with 5 mice each, subjected to a fluctuating food availability regimen designed to simulate human food insecurity. For four consecutive weeks, the experimental groups received varied, unpredictable amounts of standard chow over four days each week, followed by three days of ad libitum access (500% representing a stress recovery period). During the four-day exposure periods, mice were provided chow at 50%, 75%, 125%, and 150% of their baseline caloric consumption, in a randomly assigned order each week (Table 1). Daily weighing of chow allowed accurate assessment of food consumption at the cage level, ensuring precise monitoring of intake patterns. This regimen aimed to emulate irregular and uncertain food access characteristic of human food insecurity, where periods of sufficient food availability are interspersed with unpredictable scarcity. The control group, also consisting of two cages with 5 mice each, was maintained on consistent, ad libitum access to standard chow throughout the study. Similar to the food insecurity group, food consumption in the control group was monitored daily. This control setup provided a stable baseline against which the effects of food insecurity on tumor progression could be measured and compared. Both control and treatment groups were handled similarly to reduce variability (Table 1).

### Euthanasia and necropsy

When tumors reached 2 cm in any one direction or 2000 mm3 volume observed via ultrasound, mice were euthanized as per Unit for Laboratory Animal Medicine (ULAM) guidelines and tumors harvested. Immediately after euthanasia, tumor dimensions and weights were recorded. Portions of the harvested tumors were stored in MACS tissue freezing solution (Miltenyi Biotec) and stored at –80 °C for processing. Portions of harvested tumor were also snap frozen in liquid nitrogen and stored at -80°C for RNA and protein analysis. The remaining tumor specimen and animal carcasses were sent to the ULAM pathology core for necropsy, molecular analysis, and evaluation for metastasis.

### Blood sample collection

Approximately 1% of the mouse’s body weight, roughly 150–200 µL of blood, was collected biweekly into EDTA-coated tubes from mouse submandibular veins. To keep hormonal measurements consistent, all samples were collected 3 days post-stress recovery periods at 8:00am.

### Plasma hormonal quantification

All samples were harvested after three consecutive ad libitum feeding days, at the same time in the morning. Plasma was separated by centrifugation at 1500 × *g* for 10 min using a refrigerated centrifuge set to 4 °C. Corticosterone, adrenaline and noradrenaline, recognized as biological markers for physiological stress, were assayed from these plasma samples. Additionally, as a preliminary indicator of metastatic activity, serum levels of vascular endothelial growth factor A (VEGFA), a key promoter of angiogenesis and metastasis were quantified. All hormones were measured using commercial ELISA kits, following the manufacturer’s instructions (Abcam, CA).

### Tissue hormonal measurements

For hormonal measurements from tissue, after necropsy, xenografted tumors were rinsed with ice-cold PBS (0.01 M, pH = 7.4). Tissue specimens were weighed and minced to small samples which were homogenized in PBS containing a protease inhibitor with a glass homogenizer on ice. To further separate the cells, the suspension was sonicated with an ultrasonic cell disruptor and subjected to freeze-thaw cycles. The homogenates were then centrifuged for five minutes at 5000 × *g* to retrieve the supernatant. Hormones were measured from obtained supernatants using ELISA kit per manufacturer’s instructions (Abcam, CA). This protocol allowed for precise quantification of catecholamines, angiogenesis marker and glucocorticoids, providing insights into the biochemical stress response within the tumor microenvironment.

### Western blot

For immunoblots, 0.1 g of tumor was minced and homogenized in a RIPA buffer containing protease inhibitors and phosphatase inhibitors. Protein from tissue homogenates was measured using a BCA kit (Thermo Fisher). 25ug of protein was separated using 3–8% Tris-Acetate gels for higher molecular weight proteins and 4–20% Tris-Glycine for all other proteins (Invitrogen). For transferring, Tris-Acetate gel was incubated for 15 min in 10% methanol- containing transfer buffer before proteins were electro‐transferred to methanol-activated immobilon‐FL PVDF membranes (EMD Millipore, Billerica, MA). Membranes were then blocked with 5% milk in TBST buffer for 1 h and incubated with 1:1000 dilutions in 5% milk in TBST overnight at 4°C with primary antibodies—anti-phospho-mTOR (Cell Signaling #2974), anti-mTOR (Cell Signaling # 2983), anti‐RPTOR (Cell Signaling #2280), anti‐GBL (Cell Signaling #3274) and anti-RICTR (Cell Signaling #2114), anti-GAPDH (Cell Signaling #2118), anti-SMA Santa Cruz Biotechnology #sc-53015, anti-PARP1 (Santa Cruz Biotechnology #sc-56196), anti-MMP9 (Santa Cruz Biotechnology #sc-21733), anti-PCNA (Santa Cruz Biotechnology #sc-56). Membranes were then washed with TBST (10 min × 3), incubated with IgG HRP-conjugated secondary antibodies (Cell signaling, anti-mouse (#7076), anti-rabbit (#7074)), 1:5000 dilutions in 5% milk for 1 h at room temperature, and washed with TBST (10 min × 3). Chemiluminescence signal with ECL substrate was scanned on iBright FL1000 (Invitrogen). Signal intensities were quantified with ImageJ software (U.S. National Institutes of Health, Bethesda, Maryland, USA), and charted with Graphpad Prism version 10.0.3 (Boston, Massachusetts). All uncropped original blots are included in the manuscript under supplementary information.

### PCR array

RNA was isolated from tumor tissues using Trizol and RNeasy Universal kit as per manufacturer’s instructions (Qiagen, Michigan, USA). For cDNA synthesis RT2 First strand kit was used as per manufacturer’s protocol (Qiagen, Michigan, USA). Human Neurotransmitter Receptors (GeneGlobe ID: PAHS-060Z, Cat. No.: 330231) and Human PI3K/AKT Signaling Pathway (GeneGlobe ID: PAHS-058Z, Cat. No.: 330231) PCR array were performed. Data was analyzed using Qiagen PCR analysis portal website. Two individual samples from each group were used for replicates. Genes shown in Fig. 6A, B represent those with ≥3-fold change and a *p* value < 0.05. Expression values were normalized to internal housekeeping controls per manufacturer’s instructions.

### Immunohistochemistry

Unstained sections were cut on a rotary microtome at 4 µm thickness and mounted on glass slides. Heat-induced epitope retrieval was performed in a pH 6.2 buffer (DV2004, DIVA Decloaker, Biocare Medical) in a laboratory pressure cooker (DC2002, Decloaking ChamberTM, Biocare Medical) at temperatures alternating between 125 °C (40 seconds) and 95 °C (10 s) for a total of 60 min. Immunohistochemical staining was performed on an automated immunostainer (Biocare Intellipath, Biocare Medical). The protocol consisted of endogenous alkaline phosphatase quenching and blocking of non-specific sites (Biocare Rodent Block M, RBM961, Biocare Medical) followed by application of the primary antibody Ki67 (Cat. # ab16667, Abcam) at room temperature at the dilutions specified above. Incubation was 1 h for both primary antibodies. Detection was performed using a biotin-free polymer-based detection system (Rabbit-on-Rodent AP polymer, RMR625G, Biocare Medical) with a Fast Red chromogen (intelliPath Fast Red, IPK5017, Biocare Medical). Slides were counterstained with hematoxylin (Biocare Medical), air dried overnight, cleared in xylene, and cover slipped using a permanent mounting media. A control block with multiple types of mouse tissues was utilized as a positive control. Negative control immunostains were performed with each run using the same protocol as above with the substitution of commercially provided naïve mouse/rabbit IgG (Universal negative, IP498G20, Biocare Medical) in place of the primary antibody. Control and experimental slides were evaluated by a board-certified research veterinary pathologist (BEC) with respect to staining compartment, cell-specificity, intensity, and presence/absence of background or artifact.

### Digital slide assessment and quantification

Slides were digitized on a Leica Aperio AT2 digital slide scanner (Leica Biosystems) at a resolution of 0.25 µm/pixel (20x objective). Quantitative assessment of immunohistochemical staining was performed using the open-source program QuPath v0.4.3 (github.com/qupath/qupath). Detection parameters were optimized using the positive and negative control slides. Analysis parameters for positive cell detection are given in Table 3. All result overlays were visually checked for accuracy by a board-certified veterinary pathologist (BEC).

**Table 3:** QuPath parameters used for analysis, Ki67 immunolabeling

Image import parameters	
Image provider	Default
Image type	Brightfield, (H-DAB)
Setup parameters	
Image input	Optical density sum
Requested pixel size (µm)	0.5
Nucleus parameters	
Background radius (µm)	8
Use opening by reconstruction	True
Median filter radius (µm)	0
Sigma (µm)	1.5
Minimum area (µm^2)	10
Maximum area (µm^2)	400
Intensity parameters	
Intensity threshold	0.1
Maximum background intensity	2
Split by shape	True
Exclude DAB (membrane staining)	False
Cell expansion (µm)	2.5
Include cell nucleus	True
General parameters	
Smooth boundaries	True
Make measurements	True
Intensity threshold parameters	
Score compartment	Nucleus: DAB OD mean
Intensity threshold (chromagen)	0.1 (positive)
Single threshold	True

### Histology evaluation

Histological sections were evaluated using light microscopy at magnifications ranging from 20x to 600x by a board-certified veterinary pathologist. Lesions were assessed per International Harmonization of Nomenclature and Diagnostic Criteria (INHAND) consensus guidelines for rodent toxicologic pathology (available at https://www.toxpath.org/inhand.asp).

### Metastatic foci characterization

Foci of metastasis were characterized as “micro” if the cells did not appear to disrupt normal alveolar architecture. The overall frequency of foci was characterized as “common” or “rare” based on the subjective ease in identifying foci. Foci were additionally characterized as small, medium, or large based on the estimated number of cells per focus (≤5, 5–10, >10, respectively).

### Statistical and reproducibility

All experiments were independently repeated for a total of three trials (*n* = 10 per trial). Results were analyzed with ANOVA and Student’s *t* test using GraphPad Prism, Boston, MA, USA. All data are presented as the mean ± the standard deviation (SD) of three experiments, ns *p* > 0.05, a,*p < 0.05, b,**p < 0.01, c,***p < 0.001, d,****p < 0.0001.

### Reporting summary

Further information on research design is available in the Nature Portfolio Reporting Summary linked to this article.

## Supplementary information

Supplementary Information

Description of Additional Supplementary Files

Supplementary Data 1

Reporting Summary

## Data Availability

All uncropped original blots and raw data are included in the manuscript as Supplementary Information and Supplementary Data 1.
