# Supplementary figures and images for: Food insecurity impacts neuroblastoma pathogenesis in murine xenograft tumor models
Source: Commun Biol. 2025 Aug 31;8:1324. doi: 10.1038/s42003-025-08678-5 (PMC12399758; doi:10.1038/s42003-025-08678-5)

Supplementary figure 1: Uncropped western blots with size marker indications.

Fig. 3B

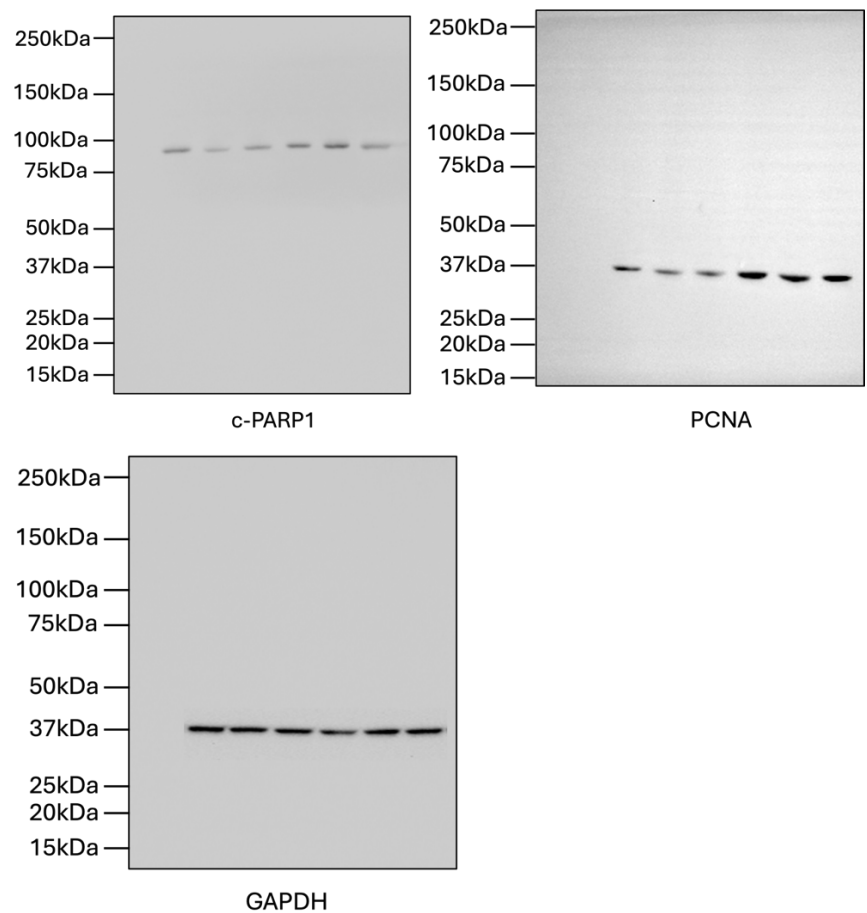

Fig. 4B

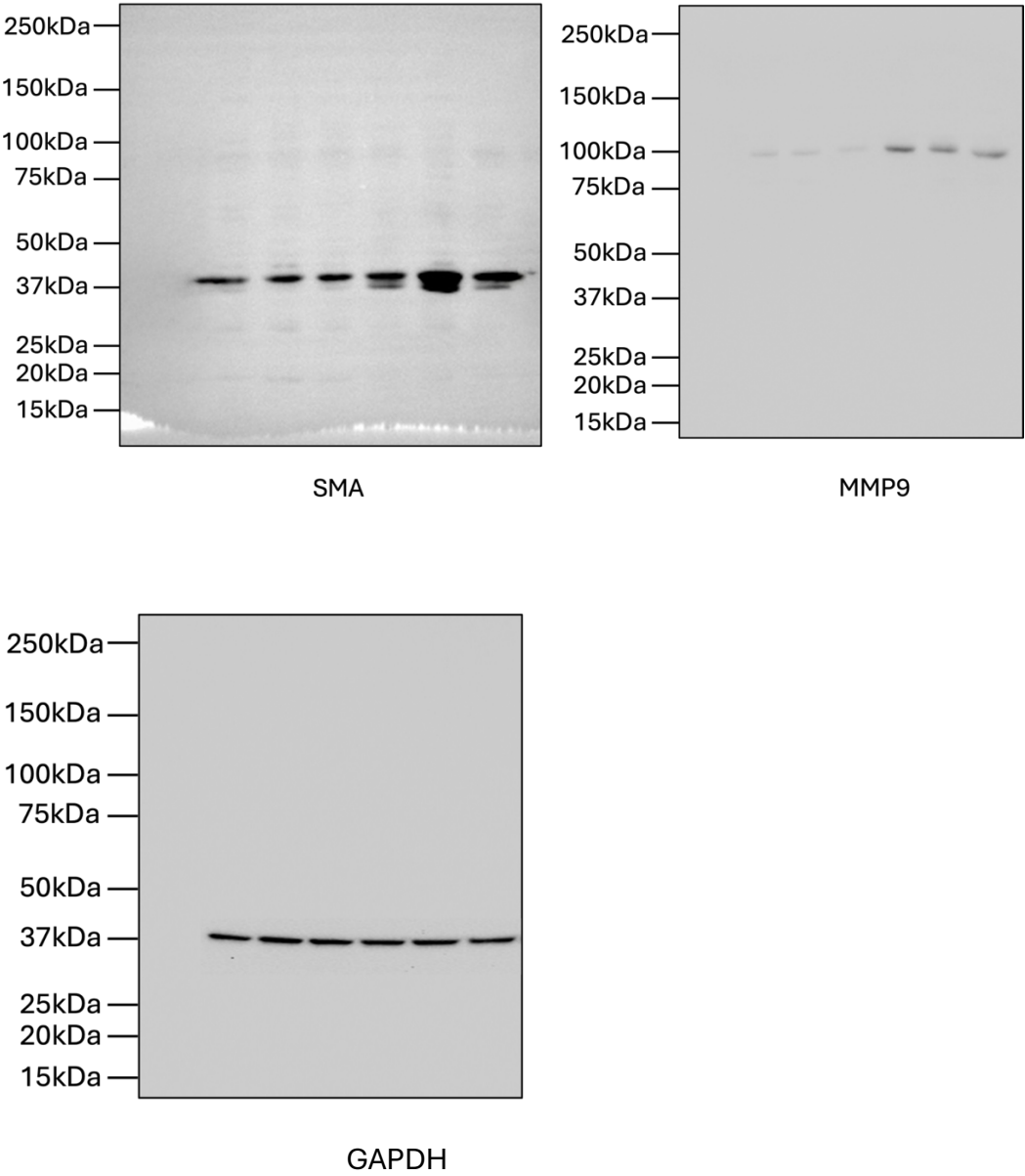

Fig. 6C

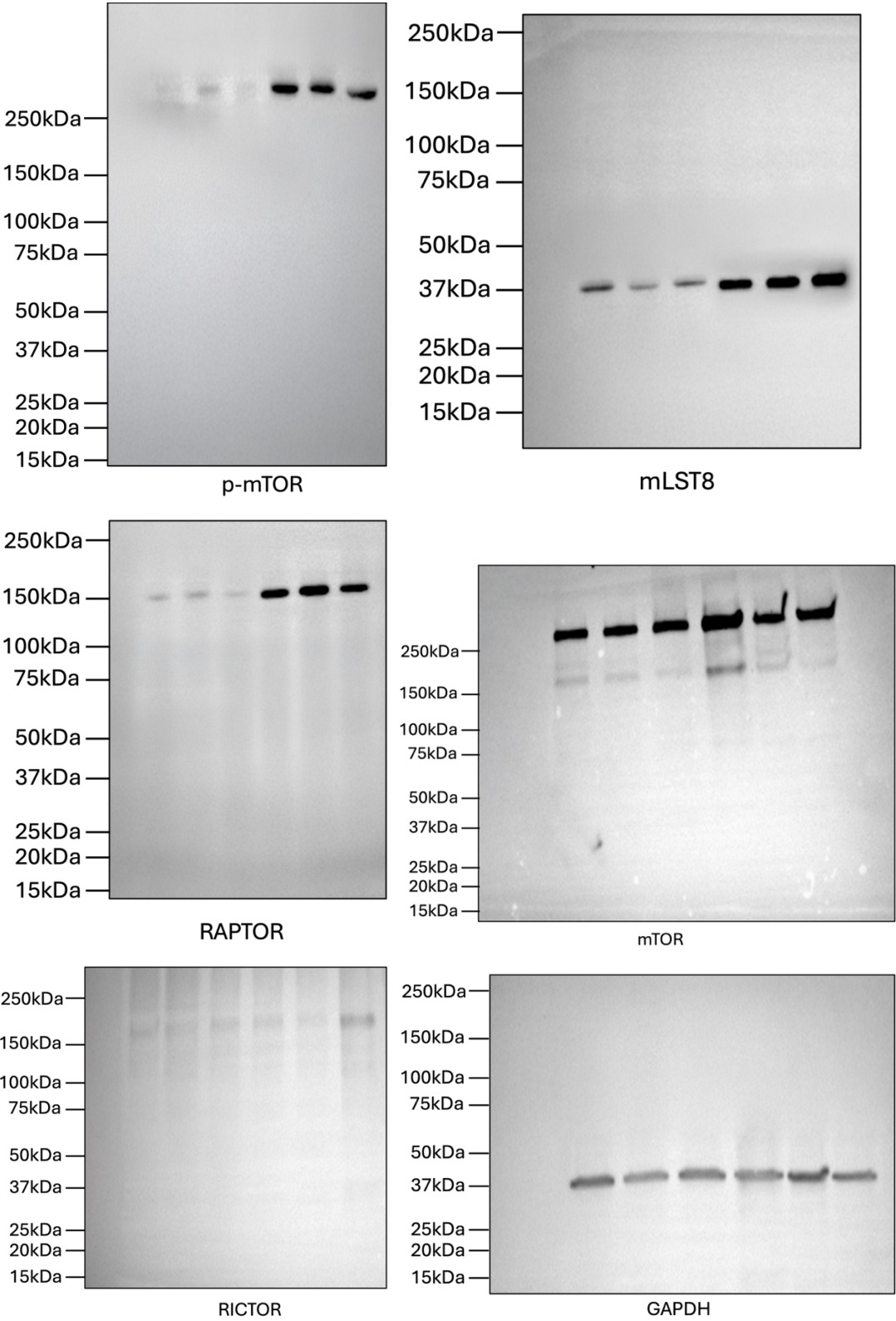

Supplement: Supplementary file 1 — Supplementary Information [file 42003_2025_8678_MOESM1_ESM.pdf]
