# Supplementary material for: Food insecurity impacts neuroblastoma pathogenesis in murine xenograft tumor models
Source: Commun Biol. 2025 Aug 31;8:1324. doi: 10.1038/s42003-025-08678-5 (PMC12399758; doi:10.1038/s42003-025-08678-5)
Supplement: Supplementary file 2 — Description of Additional Supplementary Files [file 42003_2025_8678_MOESM2_ESM.docx]

Description of Additional Supplementary Files

**File name:** Supplementary Data 1

**Description:** Raw data.
